# Management and control of coccidiosis in poultry — A review

**DOI:** 10.5713/ab.23.0189

**Published:** 2023-08-28

**Authors:** Rafiq Ahmad, Yu-Hsiang Yu, Kuo-Feng Hua, Wei-Jung Chen, Daniel Zaborski, Andrzej Dybus, Felix Shih-Hsiang Hsiao, Yeong-Hsiang Cheng

**Affiliations:** 1 Department of Biotechnology and Animal Science, National Ilan University, Yilan 26047, Taiwan; 2 Department of Ruminants Science, West Pomeranian University of Technology, Klemensa Janickiego 29, 71-270 Szczecin, Poland; 3 Department of Genetics, West Pomeranian University of Technology, 70-310 Szczecin, Poland

**Keywords:** *Eimeria* Species, Immunity, Intestinal Health, Microbiota, Poultry Production, Probiotics

## Abstract

Poultry coccidiosis is an intestinal infection caused by an intracellular parasitic protozoan of the genus *Eimeria*. Coccidia-induced gastrointestinal inflammation results in large economic losses, hence finding methods to decrease its prevalence is critical for industry participants and academic researchers. It has been demonstrated that coccidiosis can be effectively controlled and managed by employing anticoccidial chemical compounds. However, as a result of their extensive use, anticoccidial drug resistance in *Eimeria* species has raised concerns. Phytochemical/herbal medicines (*Artemisia annua*, *Bidens pilosa*, and garlic) seem to be a promising strategy for preventing coccidiosis, in accordance with the “anticoccidial chemical-free” standards. The impact of herbal supplements on poultry coccidiosis is based on the reduction of oocyst output by preventing the proliferation and growth of *Eimeria* species in chicken gastrointestinal tissues and lowering intestinal permeability via increased epithelial turnover. This review provides a thorough up-to-date assessment of the state of the art and technologies in the prevention and treatment of coccidiosis in chickens, including the most used phytochemical medications, their mode of action, and the applicable legal framework in the European Union.

## INTRODUCTION

Poultry are the primary source of animal protein around the world [1,2]. According to the USDA, 102.9 million tons of chicken meat were produced in January 2020, reflecting a 3.9%-increase over the previous year [3]. This growth is crucial, since by 2050 the world population is expected to reach approximately nine billion people, making the production of sustainable and safe protein a top priority [4]. In intensive systems, stress levels and disease prevalence are higher, mainly due to birds being kept together in large numbers and at high stocking densities [5]. Therefore, any disease that decreases the effectiveness of the aforementioned production system may pose a risk to the global food chain [6].

The poultry sector is affected by different parasitic diseases (“hidden foes”) that lead to potential chronic losses without causing apparent clinical symptoms [7]. In the United States, coccidiosis-related annual costs are estimated to be over USD 127 million [8], whereas those in China exceed USD 73 million. Coccidiosis alone accounts for 30% of the overall spending on the pharmacological control of all potential poultry diseases [9]. There are seven *Eimeria* species that have been associated with coccidiosis in chickens, with the most important being *E. tenella* in broilers. The remaining ones include *E. acervuline*, *E. brunitti*, *E. maxima*, *E. necatrix*, *E. praecox*, and *E. mitis*. They are all unique in terms of pathogenicity and each of them affects different sections of the intestines [10]. *Eimeria* infection destroys host mucosal cells, increasing cell permeability, and allowing nutrients and proteins to seep out. This, in turn, impairs digestion and absorption of proteins as well as other nutrients both of which directly contribute to the subclinical and clinical symptoms of coccidiosis [11].

Coccidiosis prophylaxis is of utmost importance to pro mote substantial growth in chicken industry and to protect the sources of financial income [12]. In Europe, it will not be possible to maintain the current level of chicken production without a comprehensive anticoccidial management program. Therefore, almost all poultry farms use antiparasitic drugs as feed additives for pullets and broiler breeders for a period of 12 to 16 weeks, as well as for broiler chickens during nearly their whole lives. This approach substantially facilitates the maintenance of the high standards of poultry safety and welfare set forth by the European Union (EU) [13]. Since 1930, a multitude of ionophore and chemical anticoccidial agents has been extensively utilized to combat these possibly hazardous parasites [14]. However, drug residues in chicken products have adverse effects on consumers’ health and lead to the emergence of drug resistance [15]. This challenge is addressed, on the other hand, by the application of coccidiostats, which are synthetic chemicals or microbial products suppressing protozoan growth. Since 1940, by Directive 70/524/EEC of November 23, they have been used in numerous farm animals to prevent, inhibit and control parasitic protozoa of the genus *Eimeria* (the most common one), *Isospora*, *Neospora*, and *Cryptosporidium*, all of which belong to the phylum Apicomplexa and cause virulent infectious diseases of the intestinal system in chickens [16]. Eleven coccidiostats are approved as feed additives in the EU and divided into two groups: synthetics (decoquinate, diclazuril, halofuginone, nicarbazin, and robenidine) and bacterial polyether ionophores (lasalocid, monensin, maduramicin, narasin, salinomycin, and semduramicin) [17]. They are usually administered from the first day of the birds’ life up to seven days before slaughter to guard against contracting the disease due to the omnipresence of oocysts. This review provides a thorough up-to-date assessment of the state of the art and technologies in the prevention and treatment of coccidiosis in chickens, including the most used phytochemical medications, their mode of action, and the applicable legal framework in the European Union.

## COCCIDIOSIS AND EIMERIA

The *Eimeria* seven-day life cycle in poultry normally occurs both outside (sporogony) and inside the host, where both sexual (schizogony and gametogenic) and asexual reproductive stages take place [18]. Fresh oocysts (capsules with protective walls shielding the parasite eggs), shed in faces at an undifferentiated (unsporulated) stage, are not infectious until they sporulate outside the host (in the environment) [19]. For the majority of *Eimeria* species, this process requires warmth and oxygen and takes 24 to 48 hours, depending on the environmental factors. Four sporocysts, each with two sporozoites, are contained in each sporulated oocyst. This is a direct cycle commencing after oral infection with active oocysts, during the infectious transmission stage. Sporocysts are released upon the mechanical action of the gizzard and sporozoites are released by the action of the bile and protein degrading enzymes in the small intestine [20].

These eukaryotic, host-specific, unicellular protozoa infiltrate the host intestinal tissue, rapidly reproducing and damaging gastrointestinal cells, thus impairing food absorption, and leading to the development of diarrhea and hemorrhage in the absence of treatment. Even in mild cases, gastrointestinal lesions induced by parasite proliferation in epithelial cells frequently facilitate other infections deteriorating animal health [21]. At least seven *Eimeria* species have been identified to parasitize intestinal epithelial cells in chickens. The pathophysiology of *Eimeria* spp. infection varies, however *Eimeria tenella*, *Eimeria necatrix*, and *Eimeria brunetti* are more hazardous and cause serious disease outbreaks in poultry. Three economically significant *Eimeria* species, *Eimeria acervulina*, *Eimeria maxima*, and *Eimeria tenella*, are the most important for broiler chickens. Following the first exposure of young animals to the infectious agent, immunity quickly develops, shielding birds against subsequent infections. Unfortunately, there is no cross-immunity among various *Eimeria* species, and recurrent occurrence of the disease may happen [22].

## METHODS USED TO CONTROL AVIAN COCCIDIOSIS

The cornerstones for the prevention and control of coccidiosis are the application of vaccines, natural feed additives, preventive anticoccidial medications, and optimal handling measures on farms [23]. Appropriate handling measures are essential at poultry farms to safeguard the health and welfare of chickens and maintain the maximum production of high-quality chicken products. Some vital measures to consider for the best management at a chicken farm are, all farm employees should receive comprehensive guidance in proper chicken handling techniques. This comprises recognizing poultry behaviours, using safe lifting and carrying techniques, and emphasizing gentle handling to reduce stress [24].

A well-designed chicken farm will enable effective and secure handling. This includes the proper lighting system, adequate ventilation, temperature management and properly designed equipment and walkways to reduce the risk of possible injury to chickens. Assure that chickens have access to clean and fresh water and sufficient diet during handling and transportation. Provision of ample electrolytes supplementation to poultry birds during stressful conditions should be considered to properly maintain their health and well-being [25]. Additionally, implement stringent biosecurity measures to avoid disease introduction and spread in the poultry farm. This specifically entails limiting access to the poultry farm, outfitting employees in proper clothing and footwear, and putting in hygienic procedures including hand washing and disinfecting tools and transportation [26]. Facility cleaning and disinfection, appropriate ventilation, and the use of clean water are some effective means of maintaining litter conditions that reduce oocyst sporulation. The application of anticoccidials to prevent disease outbreaks (prophylaxis) has been a key element in broiler chicken production [27].

### Coccidiostats

Effective anticoccidial feed additives have been conventionally used in broiler chickens and turkeys since the 1950s. According to Agri Stats Inc., during the late 1990s, 99% of broiler birds received anticoccidial treatment in one or more phases of their growth and this practice is still common around the world [28]. Meanwhile, customer preferences have evolved in different countries and currently some of the major global chicken producers, including the USA, produce as much as 60% of their broilers without anticoccidials [3]. Based on their mode of action, anticoccidial agents are classified as coccidiostats or coccidiocides. The former hinders parasite development by restricting reproduction and growth, but their effect can be significantly reduced after their elimination from the diet, which results in infection resurgence and potential disease. The latter, on the other hand, act by destroying or imposing irreparable damage to the parasites [15].

#### Mode of action of coccidiostats

Coccidiostats are classified into two groups. The first one contains polyether ionophores, which are natural compounds produced by bacteria of the family Streptomycetaceae [29]. They consist of multiple tetrahydrofuran rings conjugated into spiroketal moieties. The second group comprises synthetic coccidiostats (also known as chemicals) such as guanidines, triazines, quinolones, pyridines, alkaloids, or thiamine analogs [30]. Two modes of action (MoA) of ionophore antibiotics lead to changes in the ion concentration ratios on both sides of the cell membrane. Two antibiotic molecules dimerize under the first MoA to build a channel for cation transport through lipid bilayer [31]. The second MoA consists in the binding of cations by ionophores and their efficient transport across the cell membrane. The cations are subsequently released into the cytoplasm [32]. The cells effectively protect themselves against the aforementioned effect under physiological conditions. Ion concentrations on both sides of the cell membrane are regulated by the enzymes Ca^2+^/Mg^2+^-ATPase and Na^+^/K^+^-ATPase. As previously stated, ionophore antibiotics alter these concentrations. For example, lasalocid causes efflux of K^+^ ions while elevating the concentration of Na^+^ and Ca^2+^ within the cell [17]. The high concentration of Ca^2+^ in turn impairs mitochondrial activity, resulting in decreased energy production and cytotoxicity. Elevated Ca^2+^ levels also contribute to apoptosis due to the induction of specific endonucleases. Additionally, an inappropriate ratio of Na^+^ influx to efflux modifies the physiological ion concentrations on both sides of the cell membrane. A higher supply of energy for ATPases is essential to restore the proper functioning of the system [33]. However, the appropriate ion gradient cannot be maintained by the cell after exceeding a certain threshold and apoptosis occurs. Finally, higher levels of Na^+^ and Ca^2+^ enhance osmosis, causing cell expansion and rupture. Different chemical coccidiostats have distinct mechanisms of action. Decoquinate prevents parasite multiplication by interfering with mitochondrial electron transport [34]. Robenidine and nicarbazin are presumed to block mitochondrial energy production, but their exact mechanism of action is unknown. Halofuginone and diclazuril MoA is seldom mentioned in the literature [17].

#### Chemical coccidiostats

Currently, eleven coccidiostats, mainly synthetic chemicals and ionophores, are approved in the EU to limit disease spread, reduce parasite multiplication, and boost the immune system [21]. Ionophores eliminate parasites by preventing ions from passing through the cell membrane and altering the osmotic equilibrium. At present, they are the cornerstone of infection control. Synthetic chemicals influence parasite metabolism by blocking their physiological processes [35]. Halofuginone, robenidine, diclazuril, decoquinate, nicarbazin, toltrazuril, clopidol, ethopabate, amprolium, sulfadimethoxine, and sulfaquinoxaline are some examples of synthetic compounds [36]. Each of them has a specific mechanism of action against coccidia. Compounds of this type are referred to as “chemicals” due to their chemical composition and operate by interfering with one or more stages of the parasite life cycle [37]. They have an effect against its intestinal phases as soon as it attacks the host gastrointestinal system, being potentially more effective in the case of serious infections, but over time, resistance may appear [13].

#### Ionophores coccidiostats

Ionophores have a more complex and convoluted mode of action as compared to synthetic anticoccidials and do not exert so much selective pressure on the parasite. Furthermore, this mode of action is oriented towards sporozoites (the developmental stage of the parasite in the intestinal epithelium before host cell penetration), and does not result in the complete elimination of the parasite [38]. Instead, they allow low numbers of the parasite to survive and cause the host to acquire immunity. Currently, six ionophore anticoccidials are widely utilized in poultry production: lasalocid, maduramicin, salinomycin, monensin, narasin, and semduramicin [16].

### Vaccines

Vaccination is a crucial element of coccidiosis control strategies. It stimulates the immune response that provides protection against future *Eimeria* challenges [39,40]. This response can be immediately triggered by the production of B and T lymphocytes and lymphoid cells [23]. Vaccines are an important alternative to medications in the combat against coccidiosis. Vaccination must be carried out appropriately to be efficient and to ensure adequate protection of birds. Vaccines containing oocysts obtained from *Eimeria* strains (*E. acervulina*, *E. maxima*, *E. tenella*, *E. necatrix*, and *E. brunetti*). *E. maxima* generate the maximum immune response in the host, i.e. a single sporocyst can provoke comprehensive immune defense, and five *E. maxima* oocysts can initiate a complete immune response against infection [41]. Vaccines constitute an important component of coccidiosis prevention since they induce adaptive immune responses within 3–4 weeks, depending on host genotype, duration and frequency of infection, and parasite concentration [13]. Present day vaccination procedure is quite challenging, since there is no certainty that all poultry birds in the flocks are subjected to the same concentration of the of the coccidia. Several factors like unhygienic or insufficient administration, potentially results in suboptimal performance as compared to the preventive care given to poultry birds. The most recent advance is “*in ovo*” immunization that is applied to 18-day embryonated chicken eggs. This technique involves the precise and consistent administration of the vaccine to the embryo amniotic cavity [42]. *In ovo* inoculation is a special approach that allows us to introduce substances directly to the chicken embryo throughout the incubation period. The administration of an extensive variety of additional substances was abruptly investigated using the *in ovo* method. In 2003, the idea of *in ovo* feeding was presented with the injection of the nutrients and other natural substances that may regulate the hatchling’s gastrointestinal growth into the embryonic amnion [43].

A non-metallic vital micronutrient called selenium has the capability to modulate the immunological response in broilers exposed to *C. perfringens* and *Eimeria maxima* at 14 and 18 days after hatching, respectively. In comparison to the non-treated group, the treated group received 10 to 20 μg of selenium/egg produced and had both fewer intestinal lesions and oocysts and exhibited higher serum antibody levels against *C. perfringens* and NetB toxin. This suggests that the immune response was improved in the post-hatched period [44]. *In ovo* injection of the raffinose family oligosaccharides from the *Lupinus lutes* seeds after 12 days of incubation resulted in a 2.5 log reduction in *C. perfringens* number and an 89% decrease in the shedding of oocysts from *Eimeria* spp [45]. *In ovo* treatment of probiotics on day 18 of the incubation results in a considerable reduction in the degree of severity of macroscopic lesions induced by *Eimeria* spp. in all gastrointestinal segments as well as an improvement in the zootechnical capabilities in the broilers [46]. Sokale et al [47] employed a commercial multi-egg injector to inject 50 μL volume of commercial coccidiosis vaccine comprising *E. acervuline*, *E. maxima*, and *E. tenella* oocysts (Inovocox EMI) to Ross 708 broiler hatching eggs after 18.5 days of incubation. According to the study’s findings, administering the EMI vaccination between 18.0 and 18.8 days of incubation may safely and efficiently stimulate the broilers immune system early enough to provide protection from further coccidial assaults. Live *Eimeria* parasites administered *in ovo* can be potentially effective in preventing coccidiosis in chicken production. The *in ovo* route of administration of a live vaccine (Inovocox, Pfizer) containing oocysts of the coccidian parasites species of *Eimeria tenella*, *Eimeria acervuline*, and *Eimeria maxima* has been proven to confer protective immunity in chickens [48]. It has been documented that the DNA vaccine for *Eimeria tenella* that uses the miconeme recombinant gene (EtMIC2) stimulates protective immunity in the gastrointestinal tract against coccidiosis [49]. *In ovo* immunization with a recombinant protein subunit vaccine has additionally been shown to be successful in safeguarding chickens against coccidiosis [50]. Furthermore, according to Hamid et al [12], immunization combined with in-feed ionophores produces the best outcomes in terms of commercial broiler performance. Vaccines can be administered directly (fed as gels or added to water), topically (sprayed into the eye), or in the hatchery where chickens are raised. However, their successful implementation may be limited by their cost associated with unskilled labor and high prices [51].

The first live coccidiosis vaccine was produced more than seven decades ago. In the EU, laying pullets, commercial broilers, and replacement breeders were vaccinated for the first time in 1992, and a vaccine for commercial layers was introduced in 2000 [27]. Currently, three vaccine types are widely used under field conditions: recombinant, attenuated, and non-attenuated. Each of them contains a wide spectrum of attenuated and non-attenuated parasites [52]. Non-attenuated vaccines are effective in preventing parasite spread and have been extensively administered for about 50 years. They are used as an alternative to in-feed anticoccidials, which may be inefficient in some cases [53].

The initial choice of the *Eimeria* strain isolates can decrease the efficacy of the attenuated anticoccidial vaccines as compared to the *Eimeria* with normal life cycle. Although attenuated anticoccidial vaccines are still often employed today however, the lower level of the immune response can be increased by the potential application of the adjuvants, composed of cytokines [54]. An analysis of surface and intracellular antigens of *Eimeria* at various phases of its life cycle is necessary for producing recombinant vaccines and stimulating an efficient immune response. Problems with the identification of appropriate antigens hinders the development of synthetic vaccines [55]. However, coccidiosis in chickens can be prevented over time by rotation strategies that use both medicines and vaccines in succeeding flocks [56]. As genetic technology progresses, vaccines containing genes encoding immunomodulatory polypeptides will be developed [57].

### Natural alternatives (Plants and their components)

Alternative coccidiosis control strategies have been developed to reduce the use of veterinary medicines in the food supply chain. Natural treatments such as prebiotics and probiotics, plant and fungal extracts, and essential oils are examples of alternative therapeutic options. Typically, natural compounds influence gastrointestinal flora and the immune system rather than directly combating parasites [58].

#### Garlic

*Garlic* (*Allium sativum* L.) has been regarded as a medicinal plant for centuries. Allicin constitutes the most important organosulfur compound, accounting for over 70% of all thiosulphates and being responsible for the garlic aroma [59]. In general, allicin interacts with sulfhydryl groups of cysteine residues in thiol-containing enzymes produced by pathogenic bacteria [60].

**Figure 1:**
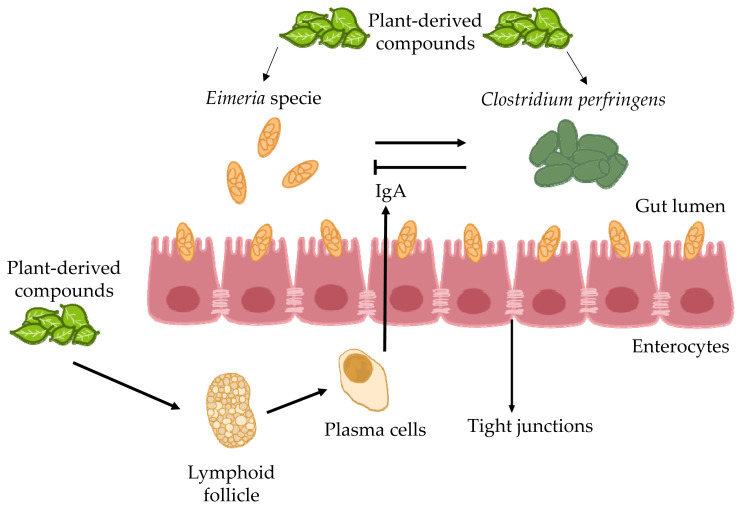
The schematic process illustrates the coordinated efforts of Gut-associated T cells and macrophages to orchestrate the immune response to anticoccidian herbal compounds in chickens.

The antioxidant and anti-inflammatory properties of garlic result from significant amounts of organosulfur compounds such as allicin, diallyl sulphide, and diallyl trisulfide. According to Kim et al [61], the potential anticoccidial effect of garlic is attributed to its immunomodulatory activity as shown in Figure 1. Aqueous garlic extract contains a high concentration of phenols, flavonoids, and other sulfur compounds [62]. Phenolic complex alters cytoplasmic membrane permeability to various cations, which affects physiological activities at the molecular level, leading to lower membrane potential, the loss of vital cellular constituents to the surrounding environment, decreased synthesis of proteins and ATP, and ultimately cellular death [63–65].

Different forms of garlic contain various active compounds with specific functions. Antioxidant activity is exhibited by powdered garlic and its aqueous extract (containing diallyl disulphide as well as phenol and flavonoids, respectively) [66], whereas the anti-inflammatory function is characteristic of powder and essential oils [67]. Moreover, diallyl trisulfide contained in the latter has antiviral properties, while the water extract also alters the permeability of the cytoplasmic membrane [68]. Finally, garlic tincture, rich in sulphuric acid, shows immunostimulatory activity and all of the above-mentioned forms are capable of inhibiting oocyst sporulation to some extent, either *in vivo* or *in vitro* [69].

#### Artemisia annua

*Artemisia annua*, a perennial plant from the Asteraceae family, is a common component of the native flora of many regions, including China, Argentina, France, Bulgaria, Hungary, Romania, Spain, Italy, and the United States [70]. The chemical constituent (artemisinin), derived from the leaves of *A. annua*, has been associated with the efficient treatment of malaria, which is a considerable problem worldwide [71]. It has been documented that the synergistic effect between plant ingredients does not decrease the incidence rate of malaria. On the other hand, *A. annua* leaves can yield 40 times higher levels of artemisinin in the bloodstream as compared to pure artemisinin [72,73]. Several recent studies on artemisinin and its derivatives have revealed that this plant may be of potential therapeutic interest for the treatment of several other diseases, such as chicken coccidiosis [74].

The number of oocysts per gram of feces in chickens ad ministered *A. annua* decreased, and the overall lesion score was 80% lower as compared to the control group. Dietary supplementation of *A. annua* in chickens resulted in reduced body weight gains but also in improved feed conversion in comparison with the control. *Artemisia annua* can be regarded as a promising candidate for the prevention of poultry coccidiosis. Furthermore, *A. annua*-supplemented broiler feeds have beneficial zootechnical and health-related properties in terms of parasitic diseases and gastrointestinal microbiota [70]. According to Almeida [75], supplementation of *Eimeria* sp. infected feed with *A. annua* dried leaves significantly reduced the number of excreted oocysts in chickens. Fatemi et al [76] also reported a decrease in the number of oocysts per gram by adding alcoholic extract of *A. annua* to poultry feed, which indicates that the potential anticoccidial effect is associated with the influence of artemisinin on oocysts. Fatemi et al [77] found that *A. annua* alcoholic and petroleum ether preparations impeded oocyst sporulation by morphological modification of the oocyst wall, whereas Del Cacho et al [78] showed that artemisinin inhibited the expression of SERCA (sarco/endoplasmic reticulum Ca^2+^ -ATPase having a vital function in transportation of calcium from the cytosol into the sarcoplasmic reticulum) in macrogametes, which had a significant impact on oocyst wall development. Furthermore, artemisinin and *A. annua* leaves promote host cell death and inhibit the inflammatory reaction. In addition, it was demonstrated that *A. annua* leaves alleviated clinical manifestations by apoptosis induction and inflammatory reaction decrease, especially in comparison with artemisinin. *A. annua* also has a substantial concentration of tannins, saponins, and flavonoids that serve as antioxidants and inhibit cellular antioxidants mediated by reactive oxygen compounds, a phenomenon observed in coccidiosis [79].

#### Biden pilosa

**Figure 2:**
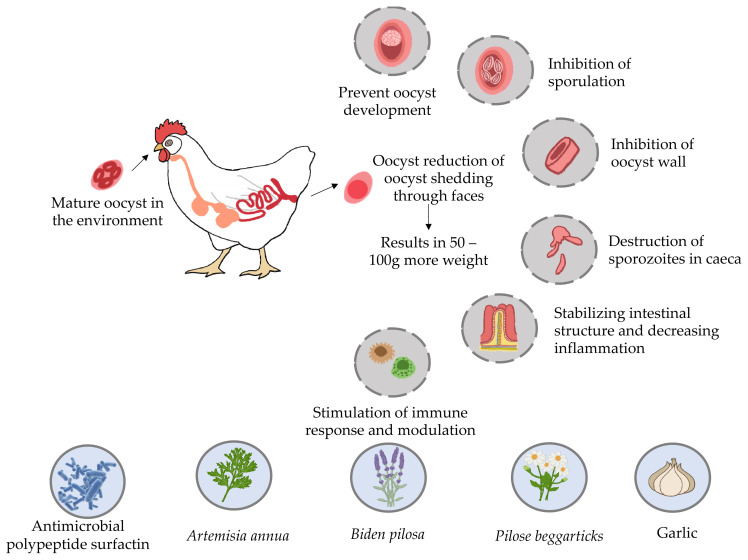
The life cycle of Eimeria, alongside an exploration of the intellectual prospects for employing natural coccidiostats.

*B. pilosa* (BP), belonging to the family Asteraceae, occurs all over the world. It has been marketed as a medicinal and culinary herb [80]. This plant has been successfully used to treat more than 41 types of infection, including protozoan and bacterial ones as well as different sorts of disorders (gastrointestinal, immunological and other) [81]. Around 200 phytochemicals such as aliphatics, flavonoids, terpenoids, phenylpropanoids, aromatics, and porphyrins, have been documented in BP. Its chemical constituents are responsible for potential therapeutic effects. In small-scale research, 68 plants, including BP, were found to have antiprotozoal activity. BP was first used as a herbal plant to treat malaria, i.e. human coccidial disease [82]. According to *in vitro* assays, BP and its active components may be successful therapeutic interventions for malaria. Polyacetylenes and flavonoids derived from BP are the key molecules responsible for its antimalarial effectiveness [83]. However, the anticoccidial properties of BP and its active ingredients require further investigation. Therefore, BP impact on *Eimeria*, the avian coccidial parasite, was explored. More recently it has been demonstrated that BP decreased the severity of *Eimeria* infection and drug tolerance in poultry. Additionally, BP positively affected symbiotic bacteria and reduced the number of pathogenic microorganisms in the gastrointestinal tract, which is the main mechanism responsible for its anticoccidial action as shown in Figure 2 [84]. Chang et al [82] found that supplementation of feed with BP at a concentration of 0.025% or higher significantly decreased the probability of *Eimeria* infection. This addition enhanced growth performance by increasing body weight gains and lowering feed conversion ratio. It also improved the anticoccidial index and reduced the number of oocysts per gram of feces, thus playing an important role in gastrointestinal pathophysiology, and decreasing mortality rate. In general, the above-described results indicate that BP can minimize the occurrence of eimeriosis in chicken production. Hence, BP can be used to effectively control this disease.

In a study comparing control with positive untreated challenged birds, feed containing probiotics (*Bacillus subtilis*, *Clostridium butyricum* and *Lactobacillus* at 5×10^8^ CFU per g), BP, and probiotic+BP increased antioxidant enzyme activity, the level of tight junction proteins and pro-inflammatory cytokines. Consequently, feeding probiotics and BP (either separately or together) to poultry seems beneficial for disease prevention and reduced intensity of *Eimeria* infection [85].

#### Oregano essential oil

The labiate family, including thyme, sage, lavender, and oregano, in particular (in the form of extract and steam-distilled oil), are the most intensively investigated plants for preventing parasitic infections in poultry. One of their most important components (polyphenols) has anticoccidial properties against chicken coccidiosis [86]. The two main phenols (carvacrol and thymol) accounting for approximately 70% to 80% of oregano essential oil, possess anticoccidial activity [87]. During *Eimeria* infection, oregano essential oil increased gastrointestinal absorption and enhanced antioxidative defense system. According to Tsinas et al [88], broilers challenged with *Eimeria acervulina* and *E. maxima* and supplemented with 300 or 600 ppm of an oregano product demonstrated decreased lesion score and without compromising growth performance. In broilers experimentally vaccinated with 50× doses of *E. acervulina*, *E. maxima*, and *E. tenella*, oregano oil supplementation at a concentration of 500 ppm decreased coccidiosis infection severity [89].

In a similar way, “functional oil” consisting of castor and cashew nut shell liquid oils added to broiler chicken feed following *E. maxima* challenge improved body weight gains and feed conversion efficiency [90]. In addition, extract from the medicinal herb Tulbaghia violacea, inhibited oocyst proliferation in *Eimeria*-infected poultry, whereas the antioxidant contained in the extract has been shown to attenuate lipid peroxidation caused by coccidial infection. It should finally be stated that herbal medicinal plants and chemicals use several metabolic pathways, including linoleic acid, estrogen, and lipoid metabolism [91].

### Bio-active compounds

#### Prebiotics

Prebiotics such as fructooligosaccharides, mannan oligosaccharides (MOS), xylooligosaccharides, and inulin, are commonly used in poultry production. They are an innovative approach to coccidiosis management and their mechanism of action is mainly based on the multiplication and activation of specialized probiotic bacteria [92]. Fructooligosaccharides and MOS have been demonstrated to modify a gut-associated inflammatory response and macrophages, which suppresses *Eimeria* infection [93]. Prebiotics exert their effect mainly through the regulation of intestinal flora by providing nutrients to favorable bacteria and stimulating their growth. They also reduce penetration of microbial pathogens into the gastrointestinal tract [94,95]. Shorter intestines of birds result in a larger amount of ingested glucose accumulating in the ceca, which, after subsequent fermentation, leads to lower pH and consequently affects *E. tenella* proliferation [96]. In the anticoccidial experiment, MOS supplementation (0.8 g/kg feed) potentially reduced the severity of cecal lesions in birds with approximately 20,000 to 30,000 sporulated oocysts of *E. tenella* [97]. In a series of studies, incorporation of MOS at a rate of 10 g/kg of feed in broiler diets resulted in lower oocyst excretion and severity of *E. acervulina* lesions. Nevertheless, other studies have not shown any beneficial effects of prebiotics on coccidiosis prevention. McCann et al [98] found that feeding 0.5 g/kg of MOS to birds did not affect severity of infection with *E. maxima*, *E. tenella*, or *E. acervuline*. Differences in the doses of *Eimeria* and MOS concentration in chicken feed were considered responsible for the discrepancies in MOS effectiveness [96].

#### Probiotics

Probiotics are defined as feed supplemented with live beneficial microorganisms (including *Bifidobacterium*, *Lactobacillus*, and *Streptococci*), yeast cultures (*Candida* and *Saccharomyces* strains), and fungi (*Aspergillus awamori*, *A. niger*, and *A. Oryza*). They have been demonstrated to decrease the susceptibility to coccidiosis by improving poultry performance, intestinal flora balance, and the immune response [5]. Feed supplementation with *Pediococcus*-based probiotics in birds infected with *E. tenella* conferred additional protection against growth delay. Ritzi et al [99] reported that supplementing broiler feed with probiotics containing *Lactobacillus salivarius*, *Enterococcus faecium*, and *Bacillus animalis* attenuated infections with *E. maxima*, *E. tenella*, and *E. acervuline* by decreasing oocyst shedding and lesion scores in the duodenum, jejunum, and ceca. When compared to the *Eimeria*-challenged positive control birds, the combination of the three aforementioned probiotic bacteria (*E. faecium*, *B. animalis*, and *L. salivarius*) at a proportion of 6:3:1 ameliorated growth parameters and improved gastrointestinal health (increasing ileal villus height and crypt depth ratio) [100]. Probiotics comprising *Pediococcus* and *Saccharomyces* strains synergistically affected the immune system reaction and decreased the shedding of *E. acervulina* and *E. tenella* oocysts [96]. *Bacillus* is another important strain that has been commonly used to treat coccidiosis in poultry. Oral administration of *Bacillus subtilis* significantly reduced *E. tenella* lesions in the ceca in comparison with the control group [101]. Similarly, eight *B. subtilis* strains contained in a direct-feed microbial product were administered to broiler chickens exposed to *E. maxima* challenge and fed the mash diet. The clinical manifestations associated with coccidiosis were reduced and the immune level was decreased by boosting cell-mediated immune responses against *Eimeria* [102]. The obtained results indicate that probiotic bacteria may occupy common receptors in the epithelium because of competing with *Eimeria* for attachment sites in the gastrointestinal tract. This competition probably prevents *Eimeria* from proliferating and shedding oocysts. However, the effectiveness of probiotics or prebiotics can be reduced by sever coccidiosis and further alternatives need to be explored [96].

#### Amino acids

Important roles of amino acids in broilers under *Eimeria* challenge

In broilers exposed to *Eimeria* spp., amino acids enhance intestinal growth, immunity, gut integrity, and antioxidant defense. Among other important amino acids, methionine, threonine, glutamine, and arginine, have received attention for their potential to mitigate the adverse effects of *Eimeria* infections in broilers [103]. Methionine is the first limiting amino acid in poultry diets produced from corn and soybeans. It is essential to produce cysteine, glutathione, taurine, carnitine, and polyamines as well as methyl group donors and sulfur donors. Because an *Eimeria* infection makes methionine harder to digest and causes more oxidative stress and inflammation inside the body, supplementing with methionine may lessen oxidative stress and enhance antioxidant defense [104]. Jankowski et al [105] revealed that broilers and turkeys supplemented with methionine had higher serum superoxide dismutase activity and total antioxidant potential. According to Castro et al [103] broilers infected with *Eimeria* spp., produced more glutathione, reduced oxidative stress, and boosted intestinal mucin synthesis as diet was supplemented with methionine.

Threonine is a crucial amino acid and is an essential con stituent of mucin for maintaining intestinal integrity and promoting spontaneous recovery during and after *Eimeria* infections as the infection damages the gastrointestinal tract [106]. According to Zhang et al [107] threonine shortage considerably enhanced the number of *Eimeria* oocysts shedding and amount of the gut leakage in the broilers receiving 25× *Eimeria* vaccine, however enhanced threonine supplementation from 0.48% to 0.96% considerably ameliorates gastrointestinal integrity and decreased oocysts shedding. Teng et al [104] documented that supplementing 0.75% threonine to low-protein diet enhanced villus height and a tight junction protein in broilers exposed to mixed Eimeria species (*E. acervuline*, *E. maxima*, and *E. tenella*). These findings suggest that supplementing broilers diet with threonine can help to improve gastrointestinal heath under coccidiosis challenge. Additionally, threonine is a vital amino acid maintaining inflammatory mechanism and regulates immunoglobulin synthesis. Dietary threonine supplementation improves IgA secretion as well as decreases pro-inflammatory cytokines including INF-*γ* and IL-1*β* [108].

#### Alkaloids

Sanguinarine (C_20_H_14_NO_4_) is a particular type of plant alkaloid. Alkaloids are nitrogen compounds particularly found in plants as secondary metabolites or natural products. Isoquinoline phenanthridine alkaloid is mostly present throughout the entire *Macleaya cordata* plant. It holds multiple potential properties including insecticidal, antibacterial, ant-inflammatory, anticancer, and immune booster [109]. As a plant derived medication, sanguinarine also has the benefits of low toxicity, little residues, and no harmful effects on the environment. As a feed supplementation, sanguinarine has been demonstrated to improve the performance of poultry. It can enhance the digestibility of nutrients and productive efficiency of laying hens fed low crude protein diet, as well as considerably safeguards laying hens under *Campylobacter hepaticus* challenges [110]. *In vitro* sanguinarine supplementation at dose rate of 1, 5, and 10 mg/L potentially inhibited coccidiosis invasion. In addition to inducing apoptosis, sanguinarine can raise sporozoites reactive oxygen species levels, lower mitochondrial membrane potential, and increase intracellular calcium ions concentrations [111].

The isoquinoline alkaloid berberine has been identified in a number of medicinal plants including *Coptis chinensis Franch*, *Cortex phellodendri*, and *Berberis asiatica*. Berberine has been linked to a magnitude of pharmacological activities such as anti-inflammatory, anti-diabetic, anti-atherosclerotic and cardioprotective properties [112]. The alteration of the intestinal microbiota composition and functionality by berberine in poultry may be correlated with its effects on the growth performance. Although, berberine increased the abundance of the phylum *Bacteroidetes* and the genus *Bacteroides* as well as decreased the *Firmicutes*, and *Clostridiales* in the gut of broilers [113]. Additionally, broilers can be treated with berberine to prevent coccidial infection and necrotic enteritis [114].

Nguyen et al [115] documented the anticoccidial advan tages associated with berberine-based supplemented diets in broilers chickens following oral infection with five *Eimeria* spp. (*E. acervuline*, *E. maximma*, *E. tenella*, *E. mitis*, and *E. praecox*). Broilers treated 0.5% berberine following *E. maxima* infection have significantly decreased fecal oocysts production.

#### Tannins

There are several forms of tannins with different molecular weights, which are classified as polyphenolic chemical compounds with the capability for precipitating proteins. The two different types of plants tannins are hydrolysable tannins with tannin derivatives (gallic acid and ellagic acid), and condensed tannins (CT) [116].

The bioavailability of tannins varies based on a number of parameters, such as the chemical derivatives of each tannin (such as gallic acid and ellagic acid), their affinity for proteins, molecular structure as well as their molecular weight. Tannin bioavailability is a crucial characteristic for their functionality and should be taken into account while addressing various challenges in poultry production [117]. Tonda et al [118] observed that supplementation of 500 mg/kg of gallnut tannic acid extract decreased total oocyst number in the excrete and lowered gastrointestinal lesions scores in broilers under *Eimeria* spp. challenges.

According to a research finding, providing chickens in fected with *Eimeria tenella* grapes seed *proanthcyanidin* extract, particularly with a high concentration in CT, as a supplement improved growth performance and reduced clinical symptoms, possibly through increasing antioxidant capacity [119]. *Eimeria maxima* infection drastically decreased broiler growth and development performance and compromised the gastrointestinal ecosystem. In broilers under *E. maxima* challenge, supplementation of tannic acid at dose of 500 to 2,750 mg/kg potentially contributed to decreased oocysts shedding, an active immune response, increased gut barrier integrity, and improved gastrointestinal impact and digestibility of nutrients. Consequently, the supplementation of tannic acid at dosage ranging from 500 to 2,750 mg/kg has the potential to act as an anti-coccidial agent and improve the gut health in broilers [120].

#### Terpenoids

The *Acacia* tree is indigenous to Egypt and is extensively grown in tropical and subtropical regions of Asia, Australia, Africa, and America. Acacia plants produce several secondary metabolites, and these secondary metabolites possess a wide range of therapeutic applications in both the prevention and treatment measure of many poultry diseases [121]. As a result, research on the biological activities of natural compounds has been conducted and developed using medicinal plants in the most effective manner [122]. Additionally, tannins, flavonoids, and saponins are known to have anticoccidial efficacy via reducing sporulation. *E. tenella* oocysts sporulation was altogether inhibited by *Acacia nilotica* aqueous extract at a concentration of 100 mg/mL, and the morphology and size of the oocysts were markedly altered, so *Acacia nilotica* can be potentially employed to prevent and cope with *Eimeria* infections [123].

#### Surfactin

Surfactin is a potent biosurfactant, produced by many strains of *Bacillus subtilis*. The biological activities of the surfactin includes antiviral, anti-mycoplasma, and antiprotozoal actions, as well as broad-spectrum potential activities against Gram-positive and Gram-negative bacteria, and fungi. Surfactin is one of the potentially successful antibiotic substitutes. Cheng et al [124] demonstrated that supplementation of *B. licheniformis* fermented product 0.1% to 0.2% to the broiler’s feed could enhance weight gain and alleviate *C. perfringens*-induced necrosis of the gastrointestinal tract. This demonstrates that the intestinal health of the broilers appeared to be improved by surfactin. According to Lee et al [125] supplementing *B. Licheniformis* fermented product 0.3% to broilers diet could enhance body weight gain. When compared to the coccidial challenge group, *B. licheniformis* fermented products improve broiler average daily growth at 21 to 35 days of age. The *B. licheniformis* fermented products treated group exhibited a higher anti-coccidia index than the coccidial challenge group. The cecal digesta of the *B. licheniformis* fermented products treated group have more genus *Lactobacillus* compared to the coccidial challenge group [126]. Cheng et al [127] reported that supplementation with *Bacillus licheniformis*-fermented products at 1 g/kg could increase average daily body weight gains in broiler chickens subjected to coccidiosis challenge. It also improved anticoccidial efficacy and modified the composition of the gastrointestinal microflora by enhancing beneficial microorganisms and inhibiting detrimental ones.

According to Yu et al [128], adding *B. licheniformis*-fermented products at 1.25 and 5 g/kg to broiler feed improved their survival rate and cecal morphology after *E. tenella* challenge. Chickens receiving 5 or 1.25 g/kg of the above-mentioned supplements had lower oocyst-count index and cecal lesion scores, respectively. Surfactin also altered sporozoite morphology and inhibited *Eimeria* oocyst sporulation. These results show that surfactin, an antimicrobial lipopeptide derived from *Bacillus licheniformis*, has anticoccidial activity both *in vitro* and *in vivo*.

Cheng et al [129] showed that *Bacillus subtilis*-supplemented feed fermented for four days had the highest surfactin level and exerted the most profound antimicrobial effect on pathogenic microorganisms such as *Clostridium perfringens*, *Staphylococcus aureus*, *Escherichia coli*, and *Salmonella typhimurium*. Dietary supplementation with *Bacillus subtilis*-fermented products containing surfactin significantly affected gastrointestinal tract morphology and stimulated the healing of ulcerated lesions in broilers infected with *Clostridium perfringens*. *Bacillus subtilis* addition may increase broiler growth and productivity, enhance bone quality, intestinal structure, and function.

## CONCLUSION

Poultry are the primary source of animal protein, contributing significantly towards meat and egg production. The demand for this type of protein rises rapidly all over the world. Each of the seven *Eimeria* species inhabits different sites of the gastrointestinal tract and causes poultry diseases ranging from subclinical enteritis to subacute mortality. The severity of coccidiosis depends on *Eimeria* species, strain, infectious dose, host genetic makeup, flock density, environmental and stress conditions, and concomitant infections. Restrictions on the use of antibiotic growth promoters and the scarcity of novel antimicrobials create an urgency to find new antibiotic alternatives. Currently, herbal products receive a great deal of attention for their potential use in the prevention and treatment of poultry coccidiosis. Research results on phytogenic substances exhibiting anti-coccidial preventive, prophylactic, or immunomodulatory activities are reviewed. However, further research is required to clarify and confirm the effectiveness of phytochemicals as promising in-feed anticoccidial agents.
